# Enhancing medical education with AI: A comparative study of various large language models in radiology

**DOI:** 10.1371/journal.pone.0360928

**Published:** 2026-11-06

**Authors:** Noha Bakhsh, Ghofran A. Ageely

**Affiliations:** 1 Department of Internal Medicine, Division of Radiology, Faculty of Medicine, King Abdulaziz University, Rabigh, Saudi Arabia; 2 Radiology Division, Department of Medicine, Faculty of Medicine, King Abdulaziz University, Rabigh, Saudi Arabia; 3 Department of Radiology, King Abdulaziz University Hospital, Jeddah, Saudi Arabia; Squad Medicine and Research (SMR), INDIA

## Abstract

**Objective:**

To compare how various Large Language Models (LLMs) perform when solving text- and image-based medical student-level radiology multiple-choice questions (MCQs).

**Methods:**

We evaluated the accuracy of seven LLMs (i.e., ChatGPT-4o, ChatGPT-5, ChatGPT-5 Plus, DeepSeek-V3.1-Terminus, Microsoft Copilot, Claude 4, and Gemini 2.5 Flash) in solving 60 text-based and 60 image-based MCQs. We compared the seven LLMs’ accuracy across question types, domains, and topics.

**Results:**

The mean accuracy across the seven evaluated LLMs was 79.9% (671/840 correct responses). Claude 4 achieved the highest observed accuracy at 86.7%, whereas Gemini 2.5 Flash and Microsoft Copilot recorded the lowest accuracy at 75.0% each. Accuracy was substantially lower for image-based questions than for text-based questions (64.5% vs. 95.2%). In the generalized estimating equations analysis, question type was significantly associated with response accuracy (Wald χ^2^(1) = 64.53, p < 0.001), as were question domain (Wald χ^2^(1) = 6.86, p = 0.009) and the question type × domain interaction (Wald χ^2^(3) = 80.26, p < 0.001). The omnibus effect of model was not statistically significant after accounting for these factors (Wald χ^2^(6) = 6.59, p = 0.361). Although Claude 4 achieved the highest observed accuracy, no pairwise model comparison remained statistically significant after Bonferroni correction.

**Conclusions:**

Large Language Models are less accurate at solving image-based than text-based radiology questions, supporting their role as adjunctive educational tools that require faculty oversight rather than independent replacements for radiology instruction.

## Introduction

In recent years, artificial intelligence (AI) has transformed numerous domains, including medicine and education. Large Language Models (LLMs), such as ChatGPT and DeepSeek, are advanced AI systems that utilize complex neural networks to process huge amounts of data for text, images, and even video generation [1]. In the context of medical education, LLMs have been able to answer questions related to learning, case simulations, decision-making, and research [2–5]. These applications not only enhance teaching but also provide learners with a more personalized and flexible learning experience [2,3].

Despite the widespread adoption of LLMs, their accuracy and reliability in medical education remains largely unexplored, and LLMs have therefore emerged as a focal point of research and discussion within the field [1–5]. Multiple-choice questions (MCQs) are among the most frequently used assessment tools in medical education, and numerous studies evaluating LLM performance on national licensing and board examination MCQs have yielded variable results [6,7]. Comparative analyses have highlighted differences in model strengths, weaknesses, and limitations, with factors such as question difficulty, subspecialty, and number of attempts shown to influence LLM performance [4,5,8–13].

LLMs encounter challenges when analysing image-based questions, and most studies have excluded questions with visual input despite their relevance to specialties such as radiology and pathology [9,11]. Although advanced LLMs have significantly improved visual input processing, leading to increased utilization among medical students, they still demonstrate lower accuracy in image interpretation compared with text-based questions [8,14].

The majority of existing studies have focused on graduate and postgraduate exams rather than medical school exams. Salvador et al. found that ChatGPT attained 61% accuracy on an image-based Radiology and Applied Physics exam for third-year medical students, compared with 78% for students overall [15]. Conversely, Ros-Arlanzón et al. found that LLMs outperformed third- and fourth-year medical students on an exam combining text- and image-based questions across infectious diseases, neurology, respiratory medicine, and cardiovascular medicine [16].

Here, we compare the performance of seven LLMs (ChatGPT-4o, ChatGPT-5, ChatGPT-5 Plus, DeepSeek-V3.1-Terminus, Microsoft Copilot, Claude 4, and Gemini 2.5 Flash) on both text- and image-based MCQs typically administered to medical students across multiple topics and learning outcome domains. Although several recent studies have benchmarked LLMs against postgraduate and licensing examinations, evidence specific to undergraduate radiology education remains scarce, and most published comparisons have evaluated text-based items only, leaving the added difficulty imposed by image interpretation largely unexamined at the medical-student level. Undergraduate radiology instruction differs from postgraduate or board-level assessment in its emphasis on foundational pattern recognition and core knowledge rather than subspecialty-level diagnostic reasoning, so findings from resident- or specialist-level LLM evaluations may not generalize to this learner population. This study addresses that gap by directly comparing seven contemporary LLMs, including newly released general-purpose models (ChatGPT-5, ChatGPT-5 Plus, and Claude 4), on a set of text- and image-based questions constructed specifically to reflect an undergraduate radiology curriculum. We hypothesized that LLM accuracy would be lower on image-based than on text-based items and aimed to determine whether this multimodal performance gap, together with variability across question domains, topics, and models, should inform how LLMs are positioned as adjunctive tools within undergraduate radiology teaching.

## Methods

### Study design

This prospective, cross-sectional comparative study evaluated the performance of large language models on a set of author-constructed multiple-choice radiology questions. The study did not involve human participants, identifiable patient data, or animal subjects. Accordingly, institutional review board approval was not required.

### Participants and materials

- **Question Selection:** We selected 60 image-based MCQs and 60 text-based MCQs representing topics taught to fourth-year medical undergraduate students. The MCQs were newly written specifically because of privacy and examination-security considerations; however, they were designed to be similar in style, structure, and difficulty to the MCQs used in fourth-year undergraduate medical radiology examinations. The questions were derived from institutional radiology teaching materials and aligned with the approved undergraduate medical curriculum. Two radiology assistant professors with more than five years of experience, trained in MCQ development and assessment, constructed the questions, following the Medical Education and Assessment Unit’s guidelines at the Faculty of Medicine, King Abdulaziz University, Rabigh, Saudi Arabia. All questions followed a single-best-answer format and included four answer options, consisting of one correct answer and three distractors. Although this process ensured curricular alignment and content relevance, the questions did not undergo formal pilot testing with students, and item-level content validity, difficulty, and discrimination indices were not formally assessed prior to use (see Limitations).
- **Question Categorization:** The questions were categorized based on type (text- or image-based), domain (knowledge or skill), and topic (introduction to radiology, respiratory imaging, cardiac imaging, gastrointestinal imaging (GIT), genitourinary imaging (GU), endocrine imaging, neurology imaging, musculoskeletal imaging (MSK), interventional radiology (IR), and radiotherapy (RT)). Categorization was performed independently by the same two radiology assistant professors who constructed the questions. Any discrepancies in categorization were resolved through discussion. If consensus could not be reached, a third senior faculty member with expertise in medical education was consulted to make the final decision. For the image-based questions, anonymized clinical images (including X-rays, CT, US, fluoroscopy, and nuclear scans) were paired with relevant questions.

### Procedures

- **Model Selection:** Seven LLMs were selected for comparison: ChatGPT-4o (OpenAI, San Francisco, CA, USA), ChatGPT-5 (OpenAI, San Francisco, CA, USA), ChatGPT-5 Plus (paid subscription; OpenAI, San Francisco, CA, USA), DeepSeek-V3.1-Terminus (DeepSeek, Hangzhou, China), Microsoft Copilot (Microsoft Corporation, Redmond, WA, USA), Claude 4 (Anthropic, San Francisco, CA, USA), and Gemini 2.5 Flash (Google DeepMind, Mountain View, CA, USA). All seven models were tested over the same 10-day period, from August 23 to September 2, 2025, using each platform’s publicly available consumer interface at the time of testing; no model version was changed or updated during this window.
- **Model Interaction:** New accounts were established in each model to avoid machine learning bias. Standardized prompts were used to input questions into each LLM, ensuring uniformity and minimizing bias. For text-based questions, the exact prompt used was: “Choose the correct answer for the following questions.” Questions were uploaded in batches of 10 MCQs per prompt. After the model generated answers for the first batch, the next batch of 10 questions was submitted until all 60 text-based questions were completed. For image-based questions, each item was submitted separately. One anonymized clinical image was uploaded directly into the model interface together with its corresponding question stem and four answer options. No image preprocessing, manual image description, or conversion of images into text was performed before submission. The exact prompt used was: “Choose the correct answer for the following question.” No additional investigator-written system prompt was used beyond the default platform settings. Each model response was accepted on the first attempt, and no retries, regeneration, or follow-up clarification prompts were permitted. Model answers were recorded exactly as generated in the initial response. The questions’ input and output data collection was conducted in one setting with a single attempt. This single-attempt, zero-shot approach (i.e., no examples, chain-of-thought instructions, or iterative refinement were provided) was chosen to capture each model’s baseline, out-of-the-box performance rather than its maximum achievable accuracy under optimized prompting; findings should therefore be interpreted as a baseline estimate under zero-shot prompting rather than a ceiling on each model’s diagnostic and reasoning potential, a point we return to in the Discussion. Because text-based questions were administered in batches of 10 while image-based questions were submitted individually, the two protocols differ in prompting strategy (e.g., potential context-window effects from batching); we treat this difference as a methodological limitation of the text-versus-image comparison rather than a controlled experimental manipulation, and address it further in the Limitations.
- Response Logging: Model responses were recorded exactly as generated and classified as correct or incorrect using a faculty-established reference answer key. Accuracy for each model was calculated as the proportion of correctly answered questions out of all questions attempted. The complete item-level response dataset underlying all analyses is provided in S1 Data.

### Statistical analysis

Statistical analyses were conducted using IBM SPSS Statistics version 27 (IBM Corp., Armonk, NY, USA). Descriptive statistics were used to summarize study variables. Categorical variables were reported as frequencies and percentages, and model accuracy was calculated as the proportion of correctly answered questions.

Because each of the 120 questions was answered by all seven LLMs, the resulting correct/incorrect responses were correlated within questions. Generalized estimating equations (GEE) with a binary logistic link, exchangeable working correlation structure, and robust standard errors were therefore used as the primary inferential analysis. The model evaluated the effects of question type (image-based versus text-based), domain (skills versus knowledge), the question type × domain interaction, and model on the probability of a correct response. Text-based questions, knowledge-domain questions, and Claude 4 were used as the reference categories. Wald χ^2^ statistics, regression coefficients, standard errors, 95% Wald confidence intervals, and p-values were reported.

Cochran’s Q test was used as a complementary omnibus analysis to compare the matched proportions of correct responses across the seven LLMs. When the omnibus test indicated an overall difference, pairwise McNemar tests were performed. Because 21 pairwise model comparisons were conducted, Bonferroni adjustment was applied, producing a corrected significance threshold of α = 0.0024. Bonferroni-adjusted p-values were also reported.

Fleiss’ kappa was calculated to quantify agreement among the seven LLMs’ binary response patterns beyond chance. Descriptive accuracy summaries were additionally generated by question type, domain, and radiology topic. Because several topic subgroups contained very few questions, including three radiotherapy questions and four interventional radiology questions, topic-level findings were treated as exploratory and were not included in the primary GEE model. All tests were two-tailed, and p < 0.05 was considered statistically significant except where adjustment for multiple comparisons was applied.

## Results

### Overall model performance

The mean accuracy across the seven evaluated LLMs was 79.9% (671/840 correct responses). Claude outperformed the other models numerically, with an accuracy of 87% (104/120). DeepSeek-V3.1-Terminus and ChatGPT-4o followed closely, with an accuracy rate of 82% (98/120) for both models. ChatGPT-5 Plus and ChatGPT-5 performed closely, with accuracy rates of 80% (96/120) and 79% (95/120), respectively. Gemini and Copilot scored the lowest, with accuracy rates of 75% (90/120) (Table 1).

**Table 1:** Performance of different LLMs on radiology undergraduate multiple-choice questions with and without imaging.

Parameters		Test score, n (%) of correct answers							Overall accuracy %
Categories		ChatGPT-4o	ChatGPT-5	ChatGPT-5 Plus	DeepSeek	Gemini	Copilot	Claude	
**All questions (n = 120)**		98 (81.7)	95 (79.2)	96 (80.0)	98 (81.7)	90 (75.0)	90 (75.0)	104 (86.7)	79.9
**Question type**	Image-based (n = 60)	40 (66.7)	38 (63.3)	39 (65)	40 (66.7)	35 (58.3)	34 (56.7)	45 (75)	64.5
	Text-based (n = 60)	58 (96.7)	57 (95)	57 (95)	58 (96.7)	55 (91.7)	56 (93.3)	59 (98.3)	95.2
**Question domain**	Knowledge (n = 49)	43 (87.8)	40 (81.6)	40 (81.6)	43 (87.8)	38 (77.6)	38 (77.5)	44 (89.8)	83.4
	Skills (n = 71)	55 (77.5)	55 (77.5)	56 (78.9)	55 (77.5)	52 (73.2)	52 (73.2)	60 (84.5)	77.5
**Question topics**	Respiratory (n = 26)	23 (88.46)	21 (80.7)	20 (76.9)	22 (84.6)	21 (80.8)	22 (84.6)	23 (88.5)	83.5
	Gastrointestinal (n = 24)	21 (87.5)	16 (66.7)	17 (70.8)	20 (83.3)	17 (70.8)	16 (66.7)	22 (91.7)	76.8
	Musculoskeletal (n = 14)	8 (57.1)	10 (71.4)	10 (71.4)	11 (78.6)	9 (64.3)	11 (78.6)	10 (71.4)	70.4
	Introduction (n = 13)	12 (92.3)	12 (92.3)	12 (92.3)	10 (76.9)	11 (84.6)	9 (69.2)	12 (92.3)	85.7
	Genitourinary (n = 12)	6 (50)	8 (66.7)	9 (75)	8 (66.7)	10 (83.3)	10 (83.3)	9 (75)	71.4
	Neurology (n = 12)	10 (83.3)	10 (83.3)	10 (83.3)	9 (75)	9 (75)	8 (66.7)	10 (83.3)	78.6
	Endocrine (n = 6)	5 (83.3)	5 (83.3)	5 (83.3)	6 (100)	3 (50)	4 (66.7)	5 (83.3)	78.6
	Cardiac (n = 6)	6 (100)	6 (100)	6 (100)	5 (83.33)	4 (66.7)	4 (66.7)	6 (100)	88.1
	Interventional radiology (n = 4)	4 (100)	4 (100)	4 (100)	4 (100)	3 (75)	3 (75)	4 (100)	92.9
	Radiotherapy (n = 3)	3 (100)	3 (100)	3 (100)	3 (100)	3 (100)	3 (100)	3 (100)	100

Table column headings use full model names where space allows; abbreviated headings are DeepSeek (DeepSeek-V3.1-Terminus), Gemini (Gemini 2.5 Flash), Copilot (Microsoft Copilot), and Claude (Claude 4).

### Performance analysis by question type

We found that LLMs performed more poorly with image-based questions than text-based questions; their average overall accuracy was 65% across all models for image-based questions and 95% for text-based questions. Claude exhibited the highest scores in both the image- and text-based categories, at 75% (45/60) and 98% (59/60), respectively. On the other hand, Gemini and Copilot exhibited the lowest accuracy for both image- and text-based questions, with Gemini having an accuracy of 58% (35/60) for image-based questions and 92% (55/60) for text-based questions and Copilot having an accuracy of 57% (34/60) for image-based questions and 93% (56/60) for text-based questions (Table 1; Fig 1).

**Fig 1:**
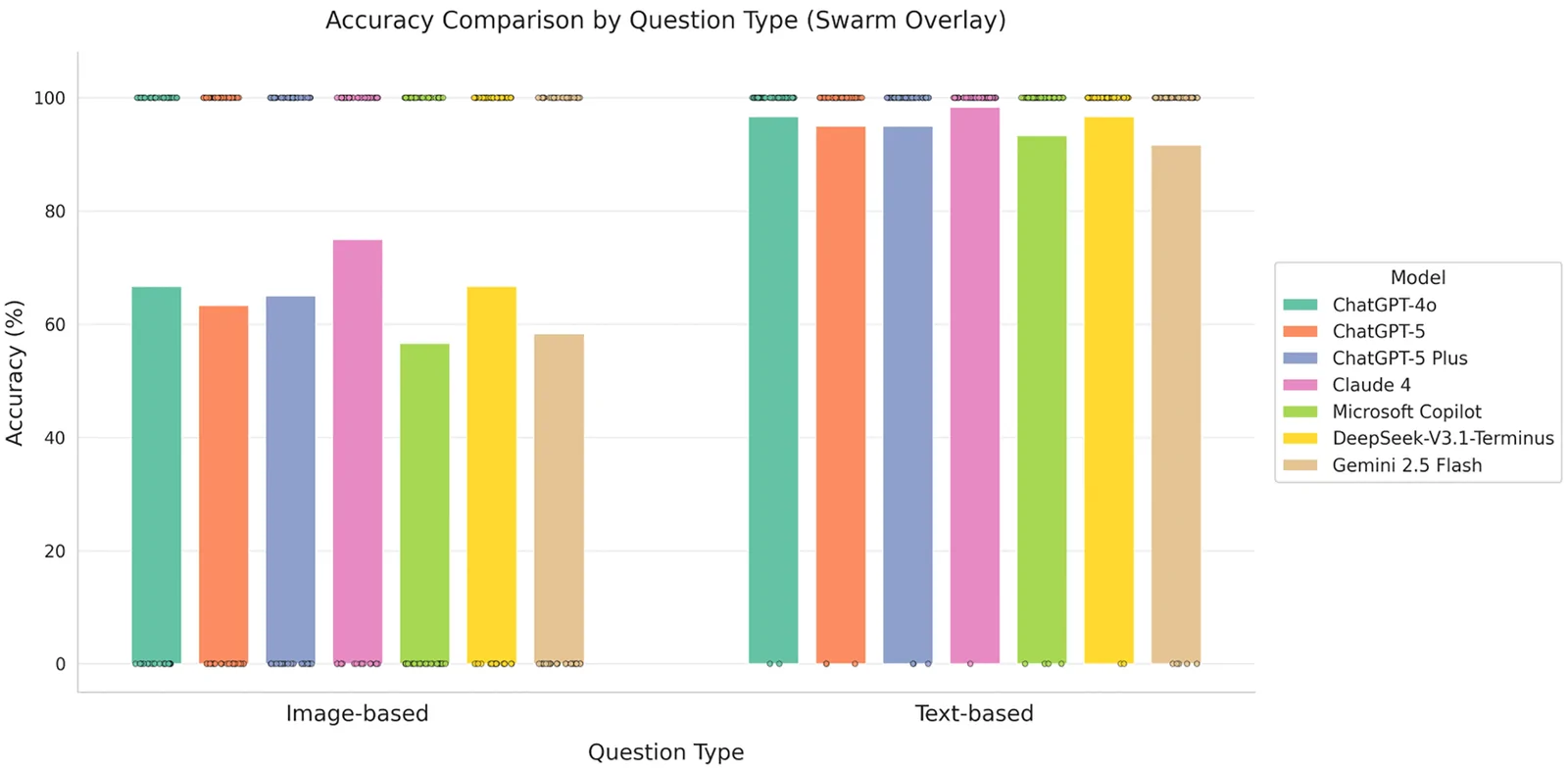
Accuracy comparison by question type (swarm overlay).

### Performance analysis by question domain

Descriptively, LLMs achieved higher accuracy on knowledge-based than on skills-based questions. Across all models, the average accuracy for knowledge-based questions was 83%; the average accuracy for skill-based questions was 77%. Claude performed the best on both knowledge- and skill-based questions, achieving an accuracy of 90% (44/49) and 85% (60/71), respectively. Conversely, Gemini and Copilot exhibited the lowest accuracy for both knowledge- and skill-based questions; each achieved an accuracy of 78% (38/49) for knowledge-based questions and 73% (52/71) for skill-based questions (Table 1).

### Comparison of LLM performance and interrater agreement

The proportion of correct responses among the seven LLMs differed significantly overall, as shown by Cochran’s Q test (χ^2^(6) = 13.58, p = 0.035). Fleiss’ kappa indicated fair-to-moderate inter-model agreement (κ = 0.4469). Model accuracy did not differ significantly within any individual question type, domain, or topic subgroup (Table 2). Because 21 pairwise comparisons were required to localize the overall difference among models, a Bonferroni-corrected significance threshold (α = 0.0024) was applied. Uncorrected pairwise McNemar tests indicated nominally higher accuracy for Claude than for Gemini (p = 0.011) and Copilot (p = 0.018); however, neither comparison remained statistically significant after Bonferroni correction for multiple comparisons (Table 3). A binary GEE model estimating model effects while additionally controlling for question type and domain likewise found no statistically significant overall effect of Model (Wald χ^2^(6) = 6.59, p = 0.361), and Bonferroni-adjusted pairwise contrasts between Claude and Gemini (p = 0.096) and between Claude and Copilot (p = 0.180) were not statistically significant. Taken together, although Claude achieved the highest observed raw accuracy, its apparent superiority over Gemini and Copilot did not withstand correction for multiple comparisons and should not be interpreted as a confirmed statistically significant difference.

**Table 2:** Cochran’s Q, df, p-value, Fleiss’ Kappa, and interpretation by question type,domain, and topics.

Category		Cochran_Q_χ^2^	df	p-value	Fleiss_Kappa
**Question type**	Image-based	5.783	6	0.4479	0.3204
	Text-based	3.99	6	0.678	0.51
**Question domain**	Knowledge	5.428	6	0.4902	0.4319
	Skills	3.55	6	0.7373	0.4506
**Question topics**	Respiratory	5.564	6	0.4737	0.2821
	Gastrointestinal	8.816	6	0.1842	0.4768
	Musculoskeletal	2.075	6	0.9126	0.3747
	Introduction	4.783	6	0.5719	0.3778
	Genitourinary	8.627	6	0.1957	0.0162
	Neurology	5.385	6	0.4955	0.1026
	Endocrine	1.838	6	0.9339	0.5522
	Cardiac	2.351	6	0.8848	0.5755
	Interventional	5.374	6	0.4968	0.4815
	Radiotherapy	–	6	–	–

**Table 3:** Pairwise McNemar test results for LLM comparison.

Model_1	Model_2	Statistic	p-value	Bonferroni-adjusted p (significant at α = 0.05?)
**ChatGPT-4o**	**ChatGPT-5**	0.2353	0.6276	1.000 (No)
**ChatGPT-4o**	**ChatGPT-5 Plus**	0.0556	0.8137	1.000 (No)
**ChatGPT-4o**	**DeepSeek**	0.0417	0.8383	1.000 (No)
**ChatGPT-4o**	**Gemini**	2.0417	0.153	1.000 (No)
**ChatGPT-4o**	**Copilot**	1.8846	0.1698	1.000 (No)
**ChatGPT-4o**	**Claude**	1.3889	0.2386	1.000 (No)
**ChatGPT-5**	**ChatGPT-5 Plus**	0.0	1.0	1.000 (No)
**ChatGPT-5**	**DeepSeek**	0.2353	0.6276	1.000 (No)
**ChatGPT-5**	**Gemini**	0.64	0.4237	1.000 (No)
**ChatGPT-5**	**Copilot**	1.0667	0.3017	1.000 (No)
**ChatGPT-5**	**Claude**	2.56	0.1096	1.000 (No)
**ChatGPT-5 Plus**	**DeepSeek**	0.0556	0.8137	1.000 (No)
**ChatGPT-5 Plus**	**Gemini**	1.1364	0.2864	1.000 (No)
**ChatGPT-5 Plus**	**Copilot**	1.3889	0.2386	1.000 (No)
**ChatGPT-5 Plus**	**Claude**	2.0417	0.153	1.000 (No)
**DeepSeek**	**Gemini**	1.8846	0.1698	1.000 (No)
**DeepSeek**	**Copilot**	2.0417	0.153	1.000 (No)
**DeepSeek**	**Claude**	1.1364	0.2864	1.000 (No)
**Gemini**	**Copilot**	0.0417	0.8383	1.000 (No)
**Gemini**	**Claude**	6.5	0.0108	0.227 (No)
**Copilot**	**Claude**	5.6333	0.0176	0.370 (No)

Note: because 21 pairwise comparisons were performed, a Bonferroni-corrected significance threshold of α = 0.0024 was applied (Methods). At this threshold, the Gemini-versus-Claude comparison (unadjusted p = 0.0108; Bonferroni-adjusted p = 0.227) and the Copilot-versus-Claude comparison (unadjusted p = 0.0176; Bonferroni-adjusted p = 0.370) are no longer statistically significant. Although Claude achieved the highest observed accuracy, pairwise differences were no longer statistically significant after adjustment for multiple comparisons (see Results).

### Generalized estimating equations analysis

The GEE model identified question type as a statistically significant predictor of response accuracy (Wald χ^2^(1) = 64.53, p < 0.001). Image-based questions were associated with lower odds of a correct response than text-based questions (B = −4.93, SE = 1.02, 95% Wald CI: −6.94 to −2.93).

Question domain was also significantly associated with response accuracy after adjustment for question type, model, and correlated observations (Wald χ^2^(1) = 6.86, p = 0.009). Skills-based questions were associated with lower odds of a correct response than knowledge-based questions (B = −2.97, SE = 1.10, 95% Wald CI: −5.13 to −0.81).

A significant question type × domain interaction was observed (Wald χ^2^(3) = 80.26, p < 0.001), indicating that the association between question type and response accuracy differed according to cognitive domain. The interaction coefficient for image-based skills questions was positive (B = 3.27, SE = 1.16, 95% Wald CI: 1.00 to 5.54), indicating that the combined effect was not adequately represented by the separate main effects alone.

After accounting for question type, domain, and their interaction, the omnibus effect of model was not statistically significant (Wald χ^2^(6) = 6.59, p = 0.361). This finding was consistent with the Bonferroni-adjusted pairwise comparisons, none of which remained statistically significant after correction for multiple testing.

Topic-level accuracy ranged descriptively from 70.4% for musculoskeletal imaging to 100% for radiotherapy. These findings were not included in the primary GEE model because of the small and unequal numbers of questions within several topic categories and should therefore be interpreted as exploratory rather than confirmatory.

### Performance across question topics

Overall, descriptive accuracy varied from 70% to 100% across the various question topics. Questions related to RT were answered correctly most frequently (n = 3, 100%); questions related to IR were answered with an accuracy of 93% (n = 4). Questions related to MSK and GU were answered correctly the least frequently: 70% and 71%, respectively. Questions related to GIT were answered correctly 77% of the time; questions related to cardiac imaging were answered correctly 88% of the time (Table 1). Several of these topic subgroups contained very small numbers of questions (e.g., n = 3 for RT and n = 4 for IR); these topic-level figures should therefore be interpreted with caution and are reported descriptively rather than as confirmed differences (see Table 4 and Discussion).

**Table 4:** Generalized Estimating Equations (GEE) analysis of predictors of a correct response.

Effect	Reference or contrast	Wald χ^2^ (df)	p-value	B	SE	95% Wald CI
Question type	Image-based vs. text-based	64.53 (1)	<0.001	−4.93	1.02	−6.94 to −2.93
Question domain	Skills vs. knowledge	6.86 (1)	0.009	−2.97	1.10	−5.13 to −0.81
Question type × domain	Image-based × skills	80.26 (3)	<0.001	3.27	1.16	1.00 to 5.54
Model, omnibus	Seven LLMs; Claude 4 as reference	6.59 (6)	0.361	—	—	—

GEE was conducted using a binary logistic link, an exchangeable working correlation structure, and robust standard errors. Reference categories were text-based questions, knowledge-domain questions, and Claude 4. B indicates the regression coefficient on the log-odds scale; SE, standard error; CI, confidence interval; GEE, generalized estimating equations; LLM, large language model. Individual model coefficients are not presented because the omnibus model effect was not statistically significant. Pairwise model comparisons are reported separately in Table 3.

## Discussion

Evaluating LLMs on undergraduate radiology MCQs is increasingly important because these models are being used to support exam-style practice and generate radiology educational and assessment content [17–19]. The performance of LLMs can differ by domain and task format, and radiology-focused evaluations have shown that model strengths coexist with clear limitations, which supports the notion that such models should be reviewed carefully when used in a teaching and assessment-preparation context [17,20,21]. One general concern documented in the literature is that LLMs can deliver incorrect responses with high confidence, a phenomenon described as hallucination [22,23]; although we did not formally assess hallucination frequency in the present study, this remains an important consideration for any deployment of LLMs in an educational context. Furthermore, outputs can vary across time and model updates, affecting reproducibility and supporting ongoing benchmarking with transparent reporting of model versions, prompts, and evaluation conditions [24,25].

### Performance by question type

The largest and most educationally important gap we observed was between text- and image-based questions: the LLMs’ average accuracy on text-based questions was 95% but only 65% on image-based questions (p < 0.001). This matters in radiology because strong performance on text-based questions can overstate competence when visual interpretation is central. This also affects how “overall” performance is interpreted, since the mean overall accuracy across the seven LLMs was 79.9%; that figure was driven disproportionately by exceptional performance on text-based items rather than by image interpretation performance, and should not be read as a single indicator of image-interpretation competence. This pattern is consistent with the text-image gap reported in a meta-analysis and in GPT-4 with vision testing on retired ACR DXIT questions [26,27]. Such a pattern has also been reported in American Registry of Radiologic Technologists (ARRT) radiography certification–style evaluations and in diagnostic radiology in-training exam comparisons, where the proportion of text-based versus image-based questions materially influences the overall accuracy [6,8].

We consider the multimodal performance gap, rather than the relative ranking of individual models, to be the principal and most educationally relevant finding of this study. Every model we tested showed a marked drop in accuracy when moving from text-based to image-based items (a 20-to-38 percentage-point decline depending on the model), indicating that the limitation lies in image interpretation broadly rather than in any single system. Claude achieved the highest observed accuracy overall (87%) and the smallest relative drop on image-based items (98% on text-based questions versus 75% on image-based questions), but this advantage did not survive statistical correction for multiple comparisons: although uncorrected pairwise McNemar tests suggested higher accuracy for Claude 4 than for Gemini and Copilot, neither comparison remained significant after Bonferroni adjustment (Table 3), and a repeated-measures model that additionally controlled for question type and domain likewise found no statistically significant overall difference among models. Accordingly, the consistent, model-agnostic reduction in accuracy on image-based items is more informative than the numerical ranking of individual models.

Several factors likely contribute to this gap, and our design cannot fully disentangle them. First, because image-based questions were presented one at a time while text-based questions were submitted in batches of ten, the two protocols differ in prompting strategy; potential context-window or batching effects mean that the text-versus-image comparison is not a fully like-for-like comparison, and this is addressed further in the Limitations. Second, we cannot rule out that some proportion of the accuracy recorded on image-based items reflects deduction from the text stem and answer options rather than genuine interpretation of the image itself; because we did not administer an image-free control version of these items, we cannot quantify this “text-leakage” effect, and we identify this as an important direction for future work rather than a measured result of the present study. Given these constraints, the observed image-text gap may overestimate the performance deficit attributable specifically to image interpretation, since some portion of the recorded gap could reflect text leakage rather than a precise, isolated measurement of visual-reasoning ability.

Güneş et al. similarly found strong performance on text-based questions but decreased performance on image-based questions in a BI-RADS study (Claude 3.5 Sonnet: 90% on text-based questions versus 59% on breast ultrasound image-related questions) [28]. On the other hand, Beşler reported a higher accuracy on image-based questions than text-based breast-imaging questions using Claude 3.5 Sonnet (i.e., 72.9% versus 59.3%) [29]. Together, these findings suggest that multimodal reliability varies by dataset, imaging modality, and use case, reinforcing the role of LLMs as adjuncts for learning rather than as primary tools for image interpretation without close human supervision.

### Performance by question domain

The descriptive accuracy was higher for knowledge-based questions than for skills-based questions (83.4% vs. 77.5%). In the adjusted GEE analysis, question domain was significantly associated with response accuracy, with skills-based questions showing lower odds of a correct response than knowledge-based questions (Table 4).

These findings suggest that question type and cognitive domain were not independent in the present dataset and should not be interpreted as separate influences on model performance. Question type and cognitive domain were not fully balanced in this item bank: image-based questions were more often skills-oriented (66.7%) than knowledge-oriented (33.3%), whereas text-based questions were more evenly split between skills (51.7%) and knowledge (48.3%) items (χ^2^ = 19.56, p < 0.001, Cramér’s V = 0.15). The significant question type × domain interaction identified in the GEE model further indicates that the association between question type and response accuracy differed according to cognitive domain, so the image-based performance deficit should not be attributed to visual interpretation alone. Earlier studies have reported mixed results regarding LLM performance across lower- and higher-order questions: some found comparable performance across cognitive levels, whereas others reported substantial declines on higher-order items, with differences in item construction, model version, prompting strategy, and the distribution of image-based questions likely explaining part of this variability. Accordingly, these findings support cautious use of LLMs for skills-oriented and image-dependent educational tasks and support the need for faculty verification.

### Performance across question topics

Descriptive accuracy varied across radiology topics, ranging from 70% to 100% (Table 1), a pattern broadly consistent with the specialty-dependent variation reported in other studies [8,27,30]. The directionally strong performance we observed in interventional radiology is similar to results reported by Pristoupil et al., although it contrasts with other studies in which interventional radiology was among the weaker domains for LLMs [27,31–33]. We also observed directionally strong cardiac imaging performance, consistent with reports of satisfactory performance in cardiovascular questions [8,32], and directionally lower performance in musculoskeletal and genitourinary imaging, concordant with reports indicating these areas may pose particular challenges in radiology-style examinations [8,27]; other studies, however, have reported higher musculoskeletal or genitourinary performance, underscoring that outcomes can shift with dataset design and the specific models tested [34,35]. Claude 4’s directionally strong performance in cardiac imaging and interventional radiology and lower performance in musculoskeletal and genitourinary imaging is in keeping with the broader multimodal variability reported by Sun et al. [36]. Because several topic subgroups were small and unequal in size (e.g., n = 3 for radiotherapy, n = 4 for interventional radiology) and the omnibus test for topic differences was not statistically significant (p = 0.7126), these topic-level patterns should be regarded as exploratory rather than confirmatory.

### Zero-shot prompting and baseline capability

All models were tested using a single zero-shot prompt, with no examples, chain-of-thought instructions, or iterative refinement. This design allowed us to compare models under equivalent, minimal-instruction conditions, but it also means that the accuracies reported here represent each model’s baseline, out-of-the-box capability rather than its maximum achievable performance. Prior literature indicates that LLM accuracy on medical examination items is highly sensitive to prompt design, and that few-shot prompting or chain-of-thought reasoning can yield substantially different results than zero-shot prompting [9]. Our findings should therefore be interpreted as a baseline estimate of model performance under zero-shot prompting rather than a ceiling on what these models can achieve in an undergraduate radiology context. Future work directly comparing zero-shot, few-shot, and chain-of-thought prompting strategies on the same question set, ideally combined with structured image preprocessing or vision-specific prompting techniques, would help determine how much of the observed image-based performance deficit is attributable to prompting strategy rather than to a fundamental limitation in visual reasoning.

### Educational implications

The substantially lower performance on image-based questions represents the most educationally important finding of this study. It indicates that although LLMs can perform strongly on text-based radiology content, their outputs regarding image-based material should not be treated as reliable without expert verification. We therefore position LLMs as adjunctive educational tools that can support revision, self-testing, and exposure to exam-style content rather than as independent or authoritative sources of radiological interpretation. Safe integration of these tools into undergraduate curricula should include structured faculty supervision, explicit instruction in critical appraisal of AI-generated content, and dedicated AI-literacy components that teach students to recognize the circumstances under which LLM accuracy is more likely to be limited.

### Performance by model cost

Access and cost also matter in education, and in our dataset the free versions of DeepSeek (82%) and ChatGPT-4o (82%) slightly outperformed the paid ChatGPT-5 Plus (80%). This finding indicates that the paid model did not demonstrate higher accuracy than the best-performing free models in this dataset. Supporting a “top-tier convergence” pattern, Adams et al. reported that the open-source Llama 3 70B performed similarly to proprietary GPT-4 Turbo and Claude 3 Opus on radiology board-style questions [37]. Other radiology benchmarks have likewise shown relatively small performance differences among leading models on written questions, although clearer declines have been observed in lower-tier systems, and DeepSeek has also outperformed older baseline chatbots on knowledge-focused examination tasks [8,34]. Overall, free or low-cost models appear capable of supporting student learning, although their outputs should still be verified against trusted educational resources.

### Contribution

Our study extends the existing literature in several ways. First, it benchmarks next-generation systems by testing newer releases, including Claude 4, ChatGPT-5, and ChatGPT-5 Plus, helping to move evaluation beyond earlier model versions. Second, it offers a clear cost-benefit message: in our undergraduate blueprint, the free models DeepSeek-V3.1-Terminus and ChatGPT-4o slightly outperformed the paid ChatGPT-5 Plus, suggesting that higher subscription cost does not automatically translate into higher accuracy for student-level learning. Finally, we observed exceptionally strong text-based accuracy overall, supporting the view that these tools may be most dependable for text-heavy radiology revision tasks.

### Limitations

This study has several limitations that warrant consideration. The 120 questions were constructed at a single institution by two faculty members and were newly written rather than formally validated: they did not undergo pilot testing with students, and item-level content validity, difficulty, and discrimination indices were not calculated. We did not include a human comparator group, such as medical students completing the same items, so the observed image-based performance deficit cannot be fully attributed to the LLMs rather than to the inherent difficulty of the items. Topic subgroups were unequal in size, ranging from three questions (radiotherapy) to twenty-six (respiratory imaging), so topic-level comparisons should be regarded as exploratory. Text-based questions were administered in batches of ten while image-based questions were submitted individually, introducing a potential context-window confound into the text-versus-image comparison, and question type was not evenly balanced with cognitive domain in our item bank, so part of the image-based performance deficit may reflect cognitive complexity rather than visual interpretation alone. Because we did not administer an image-free control version of the image-based items, we cannot quantify how much of the recorded accuracy reflects genuine image interpretation versus deduction from the text stem and answer options. Finally, incorrect responses were not subjected to formal qualitative error classification, so the study cannot distinguish image-interpretation errors, reasoning errors, and fabricated content.

### Future directions

Building on these findings, several directions merit future investigation. Validated, multi-institutional question banks that incorporate formal pilot testing and item difficulty and discrimination indices would help establish whether these performance patterns generalize beyond a single curriculum. Studies that include a human comparator cohort would clarify whether the image-based performance deficit reflects a specific limitation of LLMs or the inherent difficulty of the items themselves. A fully crossed design balancing question type and cognitive domain, together with an image-free control arm, would allow the contributions of visual interpretation, cognitive complexity, and text-leakage to be disentangled, and comparisons of zero-shot, few-shot, and chain-of-thought prompting, together with structured image-preprocessing approaches, would clarify how much of the observed multimodal gap reflects prompting strategy rather than a fundamental limitation in visual reasoning. Structured classification of incorrect responses would further help distinguish image-interpretation errors, reasoning errors, and fabricated content.

## Conclusion

Advanced AI models are highly effective for text-based radiology reasoning but remain less reliable for image-based interpretation, and this multimodal performance gap, rather than the relative ranking of individual models, is the principal finding of this study. The strong performance of free models relative to paid versions highlights an opportunity to expand equitable access to AI-supported learning. Nevertheless, the possibility of confidently delivered incorrect responses described in the broader literature, together with the performance variability we observed, underscores the need for careful governance and faculty oversight. Integrating these tools into undergraduate radiology training should position them as adjunctive educational resources that complement, rather than replace, structured faculty supervision and explicit instruction in critical appraisal, ensuring safe and effective use while preventing overreliance on these models.

## Supporting information

S1 Data Item-level LLM response dataset. Correctness (correct/incorrect) of each of the seven large language models’ (ChatGPT-4o, ChatGPT-5, ChatGPT-5 Plus, DeepSeek-V3.1-Terminus, Microsoft Copilot, Claude 4, and Gemini 2.5 Flash) responses to all 120 study questions, together with each question’s type (image- or text-based), domain (knowledge or skills), and sub-specialty classification.
(XLSX)

S1 File Graphical abstract. (PPTX)
